# Trends from 2002 to 2010 in Daily Breakfast Consumption and its Socio-Demographic Correlates in Adolescents across 31 Countries Participating in the HBSC Study

**DOI:** 10.1371/journal.pone.0151052

**Published:** 2016-03-30

**Authors:** Giacomo Lazzeri, Namanjeet Ahluwalia, Birgit Niclasen, Andrea Pammolli, Carine Vereecken, Mette Rasmussen, Trine Pagh Pedersen, Colette Kelly

**Affiliations:** 1 Department of Molecular and Developmental Medicine, University of Siena, Via Aldo Moro 2, 53100, Siena, Italy; 2 Health Scientist, Washington, DC, United States of America and Dept. of Nutritional Sciences, Pennsylvania State University, State College, Pennsylvania, United States of America; 3 National Institute of Public Health University of Southern Denmark, Øster Farimagsgade 5A, 1353, Copenhagen, Denmark; 4 Health and Wellbeing, UC Leuven-Limburg Campus Gasthuisberg Herestraat 49, 3000, Leuven, Belgium; 5 Health Promotion Research Centre, School of Health Sciences, National University of Ireland, Galway, Republic of Ireland; University of Missouri, UNITED STATES

## Abstract

Breakfast is often considered the most important meal of the day and children and adolescents can benefit from breakfast consumption in several ways. The purpose of the present study was to describe trends in daily breakfast consumption (DBC) among adolescents across 31 countries participating in the HBSC survey between 2002 to 2010 and to identify socio-demographic (gender, family affluence and family structure) correlates of DBC. Cross-sectional surveys including nationally representative samples of 11–15 year olds (n = 455,391). Multilevel logistic regression analyses modeled DBC over time after adjusting for family affluence, family structure and year of survey. In all countries, children in two-parent families were more likely to report DBC compared to single parent families. In most countries (n = 19), DBC was associated with family affluence. Six countries showed an increase in DBC (Canada, Netherland, Macedonia, Scotland, Wales, England) from 2002. A significant decrease in DBC from 2002 was found in 11 countries (Belgium Fr, France, Germany, Croatia, Spain, Poland, Russian Federation, Ukraine, Latvia, Lithuania and Norway), while in 5 countries (Portugal, Denmark, Finland, Ireland, Sweden) no significant changes were seen. Frequency of DBC among adolescents in European countries and North America showed a more uniform pattern in 2010 as compared to patterns in 2002. DBC increased significantly in only six out of 19 countries from 2002 to 2010. There is need for continued education and campaigns to motivate adolescents to consume DBC. Comparing patterns across HBSC countries can make an important contribution to understanding regional /global trends and to monitoring strategies and development of health promotion programs.

## Introduction

Daily breakfast consumption (DBC) is recommended for adults and children alike. Efforts have particularly focused on promoting and facilitating intake among schoolchildren due to associated benefits. Breakfast consumption among children and adolescents is inversely related to body mass index (BMI) and overweight in both cross-sectional [1–5] and longitudinal studies [6,7]. Regular breakfast consumption has been associated with overall dietary quality and nutrient profiles in children [1,5,6,8] and with improved cognitive performance [4,9–13]. Eating breakfast is thought to reduce snacking and consumption of energy-rich foods of poor nutrient density [1,4,5,14]. Also, regular and healthy breakfast habits in childhood track into adulthood [15–17].

Large surveys document that many children and adolescents do not regularly eat breakfast. An earlier publication from the 2005/06 Health Behaviour in School-aged Children (HBSC) study documented that in Europe between 33% and 75% of 11–15 year old adolescents reported DBC. Still, in only four countries (Netherlands, Portugal, Denmark, and Sweden) more than 70% of adolescents reported DBC [18]. US data showed that 20% of 9-13-year-olds and 32% of 14-18-year-olds in the 1999–2006 National Health and Nutrition Examination Survey (NHANES) did not eat breakfast [19]. Also, studies examining trends over time report that breakfast skipping among children and adolescents has increased over the past decades in the USA [20, 21]. In contrast, an overall increase in DBC was found in trend analyses of the Scottish HBSC study [22]. To our knowledge no study has examined trends in DBC over time across multiple countries using the same standardized methods of data collection, particularly with nationally representative samples.

Numerous factors influence breakfast consumption including socio-economic status (SES), family structure and gender. Being a child or adolescent of low SES is associated with irregular breakfast habits. This relationship exists for a range of different SES indicators, such as parental education [23,24], parental occupation [1–3], family affluence [4] and area level economic indicators [5, 6]. Moreover, while an overall increase in DBC was found in the Scottish HBSC study [22], a decrease was observed over time for older children and those from single parent families [22]; other studies have also shown DBC to be higher among two-parent families [1, 6–13]. Gender differences demonstrate that boys consume breakfast on a daily basis more often than girls [5]. Information on socio-economic correlates of breakfast consumption among children and adolescents is important for identifying adolescents and families in need of intervention and for planning initiatives that enable frequent breakfast consumption.

Studies of time trends in DBC across countries are important for identifying trends in adolescent breakfast consumption and for informing strategies for promoting breakfast consumption cross-nationally. Promoting breakfast consumption for everyone is important but those most at risk may need additional or indeed different supports. Thus, a better understanding of how young people’s breakfast habits are distributed across socio-demographic groups is important for identifying at risk groups and targeting breakfast promotion initiatives.

The purpose of this study was to analyze trends in DBC from 2002 to 2010 in adolescents aged 11 to 15 years across 31 countries participating in the HBSC survey and to identify socio-demographic (gender, family affluence and family structure) correlates of DBC.

## Methods

The data for analyses were obtained from the 2001/02, 2005/06 and 2009/10 surveys of the Health Behaviours in School-aged Children (HBSC) study. The HBSC study is a WHO collaborative study and involves an international network of research teams across Europe and North America. The overall aim of the study is to gain insight into adolescents' health and health behaviors. The populations selected for sampling are 11, 13 and 15 year olds attending school.

In total, 455,391 adolescents from national representative samples within 31 countries or regions in the three sampling waves were included. Each country follows a standardized international research protocol to ensure consistency in survey instruments, data collection and processing procedures. A clustered sampling design, with the initial sampling unit being either school class or school was used. Participating countries were required to include a minimum of 95% of the eligible target population within their sample frame. The recommended sample size for each of the three age groups was approximately 1,500 students, assuming a 95% confidence interval of +/- 3 percent around a proportion of 50 per cent and allowing for the clustered nature of the samples. More detailed information about the study is provided elsewhere [25,26].

The survey instrument is an internationally standardized self-report questionnaire that is administered in the classroom by trained personnel, teachers, or school nurses and whose completion takes approximately 50min. Parental written informed consent to participate was obtained before administration. Student participation was voluntary and anonymity and confidentiality of the data were ensured. In Italy ethical approval was granted by the Italian Higher Institute of Health. All participating countries within the HBSC network must adhere to ethical guidelines and principles as described in the study protocol. Adherence to protocol requirements is managed by the HBSC International Coordinating Centre in Bergen, Norway [26].

### Measures

#### Outcome variable

To assess DBC, adolescents were asked to indicate how many days they generally have breakfast (defined as having more than a glass of milk or fruit juice) on schooldays and on weekends. Response categories were "never" to "five days" for schooldays, and "never" to "two days" for the weekend. These responses were summed to a total range of days eating breakfast (0–7 days a week) and dichotomized into "daily breakfast consumption" (7 days) versus "less than daily" (0–6 days).

#### Explanatory variables

Socio-economic position: The Family Affluence Scale (FAS) was used to assess socioeconomic position. A sum score was constructed from the following four items: “Does your family own a car, van or truck” (No/Yes one, Yes two or more (0–2 points)). “Do you have your own bedroom for yourself? (No/Yes (0–1 points)). “During the past twelve months, how many times did you travel away on holiday (vacation) with your family?” (Not at all/ Once / Twice / More than twice (0–3 points respectively)) and “How many computers does your family own? (None/ One/ Two/ More than two (0–3 points). For each country, the FAS score was divided into low (0–3 points), medium (4–6 points) and high FAS (7–9 points) [26].

Family structure: Based on student reports of who they live with most of the time, family structure was categorized into living with two parents, one parent or with “others”.

#### Statistical analyses

Multilevel logistic regression analyses for the binary outcome variable "daily versus non-daily breakfast consumption" were conducted separately by country on data pooled across surveys. The independent variables in the model were family affluence, family structure and year of survey. Pseudo likelihood estimation methods, the Binomial probability distribution and the Logit link function were used. Only countries with complete information and association daily-daily ≥ 50% were included. This means that the predictive ability of the model to correctly classify a person who eats breakfast every day is more than 50%. Otherwise it would mean that the model fails to properly classify these subjects and thus the model is not considered good.

The estimates were performed for each country separately with adolescents nested within classes and classes within schools (three-level random intercept model) and adjusted for age category. The Bonferroni’s sequential test was used to compare DBC between categories of the variables considered. OR and CI (95%) were calculated and the Wald test was used to identify significant parameter estimates; the value of Bayesian Information Criterion (DIC) was used as a measure of model fit. Fixed and random parameter estimates for the model were tabulated, where fixed estimates were defined as the average effect across the entire population of schools, classes and individuals, while the random estimates described how these varied at each level (school and classes) [27]. All independent variables are presented as dummy indicator variables, contrasted against a base category. P-values < 0.05 were considered significant. The statistical package “Generalized Linear Mixed Models” in SPSS software (v.22.0) was used for all analysis.

## Results

**Fig 1:**
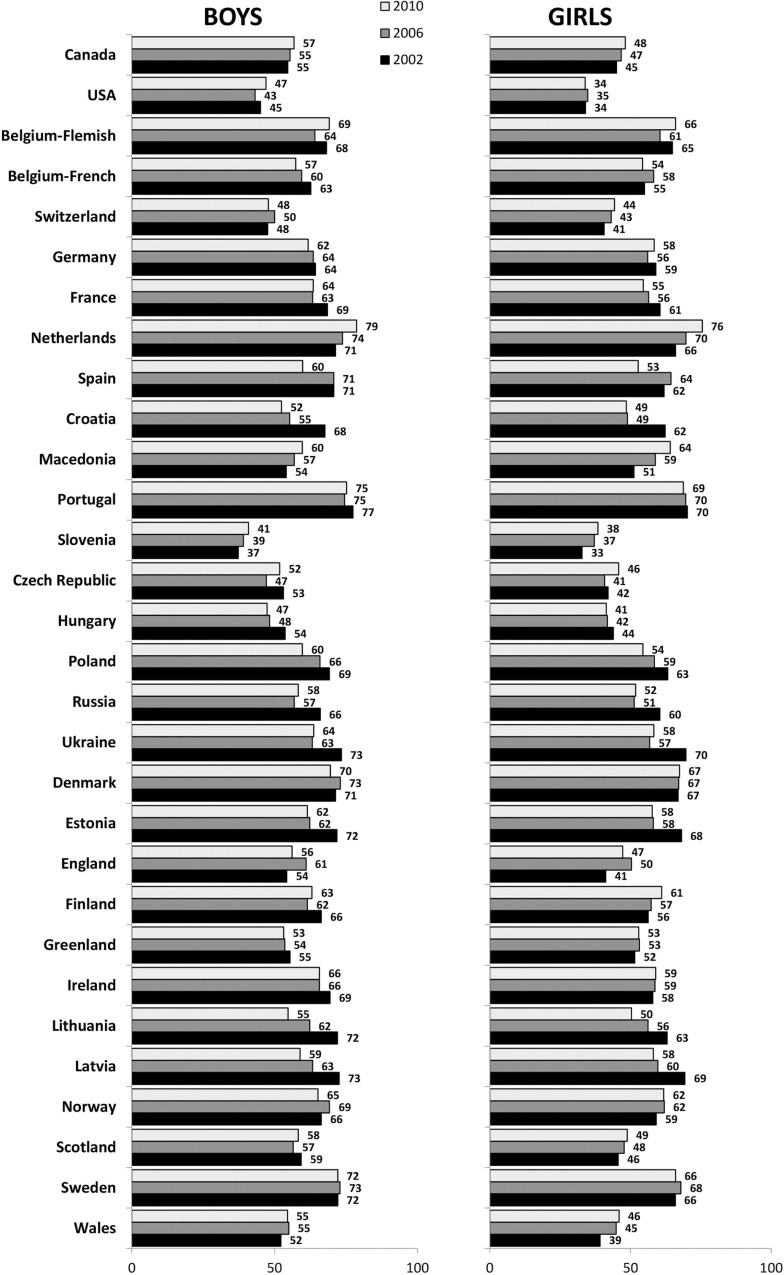
Daily breakfast consumption among 11, 13 and 15 years old by country, survey year and gender (%)

Data on 455,391 adolescents in the 31 countries or regions were included (Table 1). In total, boys constituted 49.1% and girls 50.9% with only small differences between countries. DBC ranged between 37.8% (Slovenia) to 72.6% (The Netherlands). Among boys, DBC ranged from 39.3% (Slovenia) to 75.6% (Portugal) and among girls between 36.4% (Slovenia) to 70.7% (The Netherlands) (Table 1 and Fig 1).

**Table 1:** Country specific study populations by socio-demographic characteristics, daily breakfast consumption and survey year.

	Gender (%)		Family Affluence (%)			Family Structure (%)			Breakfast (%)	Survey year (absolute frequency)			
	Boy	Girl	High	Medium	Low	Two	Single	Other	Daily^§^	2002	2006	2010	Total
**Non-European countries**													
Canada	47.8	52.2	20.8	41.9	37.4	67.2	26.8	6.0	51.5	4361	5930	15919	26210
United States	49.0	51.0	21.6	38.8	39.6	58.0	36.8	5.2	40.8	5025	3892	6274	15191
**Central European countries**													
Belgium—FI	49.3	50.7	17.2	36.4	46.4	75.9	19.1	5.0	65.6	6289	4311	4180	14780
Belgium—Fr	49.7	50.3	17.5	33.9	48.6	67.9	28.8	3.3	57.9	4323	4476	4012	12811
France	49.7	50.3	18.7	39.2	42.1	74.8	22.6	2.5	61.3	8185	7155	6160	21500
Germany	49.5	50.5	18.4	37.6	43.9	74.5	21.9	3.6	60.4	5650	7274	5005	17929
Netherlands	50.3	49.7	22.7	41.9	35.4	80.2	12.9	6.8	72.6	4268	4278	4591	13137
Switzerland	49.6	50.4	20.2	41.0	38.8	79.2	19.1	1.8	45.6	4679	4621	6678	15978
**Southern European countries**													
Croatia	48.9	51.1	17.5	36.1	46.3	87.8	8.0	4.2	55.0	4397	4968	6262	15627
Macedonia	49.8	50.2	27.0	37.8	35.2	88.8	5.5	5.7	57.3	4161	5281	3944	13386
Portugal	47.6	52.4	28.3	36.5	35.2	78.1	15.2	6.7	72.3	2940	3919	4036	10895
Slovenia	50.4	49.6	17.7	38.9	43.4	83.8	14.1	2.1	37.8	3956	5130	5436	14522
Spain	48.6	51.4	32.1	20.9	46.9	82.2	14.0	3.7	64.2	5827	8891	5040	19758
**Eastern European countries**													
Czech Republic	49.0	51.0	18.0	35.9	46.1	70.5	27.3	2.2	46.6	5012	4782	4425	14219
Hungary	46.5	53.5	20.3	34.2	45.5	73.8	23.6	2.7	45.7	4164	3532	4864	12560
Poland	49.2	50.8	19.0	34.7	46.2	83.5	14.5	2.1	62.4	6383	5489	4262	16134
Russian Federation	47.7	52.3	18.9	36.1	45.0	50.5	18.8	30.6	57.7	8037	8231	5174	21442
Ukraine	49.6	50.4	19.0	36.3	44.7	74.5	22.6	3.0	63.5	4090	5069	5890	15049
**Northern European countries**													
Denmark	48.2	51.8	22.4	41.5	36.1	61.3	18.7	20.0	69.2	4672	5741	4330	14743
Scotland	49.9	50.1	17.5	36.7	45.8	66.7	22.1	11.2	52.7	4404	6190	6771	17365
Wales	50.5	49.5	16.0	38.2	45.9	63.6	31.4	4.9	48.9	3887	4409	5454	13750
England	48.5	51.5	19.7	37.6	42.6	67.1	28.7	4.3	51.1	6081	4783	3524	14388
Estonia	49.1	50.9	23.4	34.9	41.7	67.6	30.6	1.8	63.1	3979	4484	4236	12699
Finland	48.8	51.2	32.3	21.5	46.1	71.9	25.6	2.5	61.0	5388	5249	6723	17360
Greenland	47.3	52.7	21.5	34.4	44.1	50.8	31.4	17.7	53.2	891	1366	1207	3464
Ireland	50.3	49.7	21.9	41.9	36.2	76.4	16.9	6.7	62.5	2875	4894	4965	12734
Latvia	47.9	52.1	17.5	33.7	48.8	64.5	30.9	4.6	63.1	3481	4245	4284	12010
Lithuania	51.4	48.6	15.9	36.9	47.2	72.2	17.8	10.0	60.0	5645	5632	5338	16615
Sweden	49.9	50.1	23.4	21.8	54.8	73.8	16.5	9.7	69.4	3926	4415	6718	15059
Norway	50.9	49.1	15.0	43.4	41.7	70.3	23.4	6.3	63.9	5023	4711	4342	14076

^§^Percentage of children who report to consume breakfast every day across each of the 3 rounds of the survey (% on total N)

The mean FAS score ranged from 3.90 (SD = 1.80) in Ukraine to 6.68 (SD = 1.65) in Norway. The proportion of children living in high FAS homes ranged from 15.9% (Norway) to 28.3% (Portugal); Medium FAS from 21.5% (Finland) to 43.5% (Norway); and Low FAS from 35.2% (Portugal and Macedonia) to 48.8% (Latvia). Distributions of DBC by FAS showed that in the High FAS group DBC ranged from 42.3% (Slovenia) to 73.1% (The Netherlands); from 38.7% (Slovenia) to 70.1% (The Netherlands) in the Medium FAS group; and from 35.1% (United States) to 66.8% (Portugal) in the Low FAS group.

For family structure, the proportion of children living with both parents ranged from 50.8% (Greenland) to 88.8% (Macedonia) while between 5.5% (Macedonia) and 31.5% (Greenland) lived in a single parent household. Distribution of DBC by family structure showed that in all countries, children in two-parent families were more likely to report DBC compared to single parent families. Children in “other” types of family structures in most countries had rates of DBC in between children from two parent and single parent families. In two-parent families, DBC ranged from 44.4% (United States) to 75.5% (the Netherlands); while in single-parent families, the range was from 34.5% (Slovenia) to 66.4% (Portugal). Reported DBC for children in “other” family structure ranged from 38.7% (United States) to 68.7% (Portugal).

Distributions of DBC by survey year revealed that in many countries the proportion of adolescents reporting DBC was lower in 2010 compared to 2002. In 2002, DBC ranged from 36.7% (Slovenia) to 67.6% (Denmark); in 2006 it ranged from 38.6% (United States) to 70.6% (The Netherlands); and in 2010 from 40.5% (Slovenia) to 74.1% (the Netherlands) (Table 2).

**Table 2:** Country specific daily breakfast consumption by family affluence, family structure and survey year.

	Family Affluence (%)			Family Structure (%)			Survey year (%)		
	High	Medium	Low	Two-parent	Single	Other	2002	2006	2010
**Non European countries**									
Canada	51.6^a^	50.0^b^	47.4^a^^,^^b^	54.8^c^	41.9^c^^,^^d^	52.3^d^	47.7	50.3	51.0
United States	44.4^a^	40.0^b^	35.1^a^^,^^b^	44.4^c^	36.3^c^	38.7	38.3^d^	38.6	42.4^d^
**Central European countries**									
Belgium—FI	66.1^a^	63.9^b^	58.0^a^^,^^b^	67.9^c^	57.8^c^	62.2	-	60.1^d^	65.4^d^
Belgium—Fr	62.7^a^	59.9^b^	51.4^a^^,^^b^	62.0^c^	54.5^c^	57.6	60.7^d^	59.1^e^	54.4^d^^,^^e^
France	63.4^a^	62.1^b^	55.5^a^^,^^b^	63.7^c^	56.3^c^^,^^d^	61.0^d^	64.3^e^	59.4^f^	57.3^e^^,^^f^
Germany	65.0^a^	60.1^b^	52.8^a^^,^^b^	64.5^c^	56.1^c^	57.5	62.6^d^	58.1	57.5^d^
Netherlands	73.1^a^	70.1^b^	66.2^a^^,^^b^	75.5^c^	66.2^c^	67.4	64.4^d^	70.6^e^	74.1^d^^,^^e^
Switzerland	46.5^a^	42.2^b^	39.0^a^^,^^b^	48.8^c^	36.6^c^	42.4	-	43.0	42.1
**Southern European countries**									
Croatia	55.9^a^	54.9^b^	51.5^a^^,^^b^	56.6^c^	51.6^c^	54.0	63.8^d^	50.3	47.8^d^
Macedonia	48.9	53.3	52.2	57.8^a^	51.2^a^	45.4	45.4^b^	52.1^c^	56.9^b^^,^^c^
Portugal	72.1^a^	71.1^b^	66.8^a^^,^^b^	74.6^c^	66.4^c^	68.7	71.3	69.1	69.6
Slovenia	42.3^a^	38.7^b^	36.2^a^^,^^b^	38.6^c^	34.5^c^^,^^d^	44.3^d^	36.7^e^	39.9	40.5^e^
Spain	64.3^a^	61.4^b^	57.9^a^^,^^b^	65.7^c^	56.5^c^^,^^d^	61.3^d^	64.0^e^	65.9^f^	53.5^e^^,^^f^
**Eastern European countries**									
Czech Republic	47.1^a^	45.8^b^	43.2^a^^,^^b^	49.8^c^	41.2^c^	45.1	47.6	42.9^d^	45.6^d^
Hungary	48.3^a^	48.6^b^	43.0^a^^,^^b^	47.3^c^	43.3^c^	49.3	49.2^d^	45.8	44.8^d^
Poland	64.3^a^	63.5^b^	59.1^a^^,^^b^	63.8^c^	56.4^c^^,^^d^	66.5^d^	67.2^e^	62.6^f^	56.9^e^^,^^f^
Russian Federation	56.1	56.3	57.0	57.2^a^	53.4^a^^,^^b^	58.7^b^	62.9^c^	54.2	52.1^c^
Ukraine	64.0^a^	63.9^b^	61.3^a^^,^^b^	65.4^c^	63.0^c^	60.8	70.1^d^	58.7	59.9^d^
**Northern European countries**									
Denmark	71.2^a^	68.7^b^	63.6^a^^,^^b^	72.7^c^	62.7^c^^,^^d^	68.0^d^	67.6	69.2	67.0
Scotland	52.2^a^	50.6	49.1^a^	55.8^b^	46.4^b^	49.5	49.8	50.4	51.6
Wales	47.4^a^	47.3^b^	43.1^a^^,^^b^	52.3^c^	43.0^c^	42.6	43.1^d^	47.4	47.3^d^
England	52.2^a^	49.8^b^	46.7^a^^,^^b^	55.3^c^	46.2^c^	47.2	46.2	53.0	49.5
Estonia	60.5	59.2	58.3	62.1^a^	55.7^a^	60.2	-	59.8	58.8
Finland	60.7^a^	59.2^b^	56.8^a^^,^^b^	65.0^c^	52.8^c^^,^^d^	58.6^d^	59.7	57.5	59.5
Greenland	53.8	50.5	49.5	53.4^a^	43.7^a^^,^^b^	56.7^b^	-	-	51.3
Ireland	63.7^a^	62.2^b^	57.7^a^^,^^b^	64.9^c^	54.3^c^^,^^d^	64.2^d^	61.1	61.7	60.9
Latvia	60.8^a^	62.9	64.8^a^	64.7^b^	59.9^b^	63.9	69.5^c^	60.4	58.3^c^
Lithuania	59.7^a^	59.7^b^	56.9^a^^,^^b^	61.6^c^	55.6^c^	59.0	65.9^d^	58.8^e^	51.2^d^^,^^e^
Sweden	67.9^a^	66.7	65.5^a^	72.9^b^	64.4^b^	62.4	67.1	67.3	65.7
Norway	47.4^a^	47.3^b^	43.1^a^^,^^b^	52.3^c^	43.0^c^	42.6	43.1^d^	47.4	47.3^d^

^a,b,c,d,e,f^ Bonferroni’s sequential test, p<0.05. The same index in the rows for each variable identifies the significant difference

**Associations between DBC and socio-demographic factors**. A total of 22 countries or regions had complete data to examine associations between DBC and socio-demographic factors. DBC was associated with being a child of a two-parent family with an OR between 1.17 (95% CI: 1.08–1.26) in the Russian Federation to 1.78 (955 CI: 1.63–1.93) in Norway, compared to being a child of single parent family. In most countries (n = 19), DBC was associated with being a child living in a family with high FAS score compared to being a child living in a family with low FAS status. OR ranged from 1.12 in Ukraine (95% CI: 1.02–1.24), to 1.72 (95% CI: 1.54–1.91) in Germany. In Macedonia and Russia no association between DBC and family affluence was found (Table 3).

**Table 3:** Logistic regression analyses (OR, 95% CI)* of the association between daily breakfast consumption and family affluence, family structure and survey year, separately by country.

	Family Affluence		Family Structure		Survey year	
	High	Medium	Two-parent	Other	2002	2006
	Vs	Vs	Vs	Vs	Vs	Vs
	low	low	single	single	2010	2010
**Non-European countries**						
Canada	**1.18**	**1.10**	**1.67**	**1.49**	**0.80**	0.97
	**(1.09–1.27)**	**(1.04–1.17)**	**(1.57–1.78)**	**(1.30–1.71)**	**(0.72–0.89)**	(0.88–1.07)
**Central European countries**						
*Belgium—Fl	**1.43**	**1.30**	**1.52**	1.15	-	**0.86**
	**(1.25–1.64)**	**(1.16–1.45)**	**(1.35–1.70)**	(0.89–1.50)		**(0.77–0.97)**
Belgium—Fr	**1.58**	**1.41**	**1.35**	1.13	**1.24**	**0.20**
	**(1.41–1.77)**	**(1.29–1.54)**	**(1.24–1.47)**	(0.90–1.43)	**(1.11–1.39)**	**(1.08–1.34)**
France	**1.37**	**1.31**	**1.34**	1.17	**1.37**	**1.09**
	**(1.26–1.49)**	**(1.23–1.40)**	**(1.25–1.43)**	(0.96–1.43)	**(1.26–1.48)**	**(1.01–1.18)**
Germany	**1.72**	**1.38**	**1.41**	1.00	**1.21**	1.06
	**(1.54–1.91)**	**(1.27–1.50)**	**(1.29–1.54)**	(0.82–1.23)	**(1.04–1.41)**	(0.96–1.16)
Netherland	**1.41**	**1.21**	**1.60**	1.10	**0.65**	**0.80**
	**(1.26–1.59)**	**(1.10–1.33)**	**(1.41–1.81)**	(0.90–1.34)	**(0.57–0.74)**	**(0.71–0.90)**
**Switzerland	**1.35**	**1.13**	**1.59**	1.24	**-**	1.01
	**(1.21–1.51)**	**(1.03–1.24)**	**(1.44–1.76)**	(0.88–1.74)		(0.93–1.11)
**Southern European countries**						
Croatia	1.09	1.04	**1.21**	1.11	**1.85**	1.06
	(0.99–1.20)	(0.97–1.12)	**(1.07–1.37)**	(0.90–1.38)	**(1.67–2.06)**	(0.96–1.17)
Macedonia	0.95	1.04	**1.30**	0.80	**0.63**	**0.82**
	(0.85–1.05)	(0.95–1.14)	**(1.09–1.55)**	(0.63–1.02)	**(0.54–0.74)**	**(0.71–0.95)**
Portugal	**1.26**	**1.22**	**1.50**	1.07	1.02	1.01
	**(1.12–1.41)**	**(1.10–1.36)**	**(1.33–1.69)**	(0.87–1.31)	(0.90–1.16)	(0.90–1.14)
Spain	**1.28**	**1.13**	**1.46**	1.17	**1.43**	**1.54**
	**(1.19–1.38)**	**(1.04–1.23)**	**(1.34–1.60)**	(0.98–1.40)	**(1.29–1.59)**	**(1.39–1.70)**
**Eastern European countries**						
Poland	**1.22**	**1.19**	**1.37**	**1.51**	**1.57**	**1.30**
	**(1.11–1.34)**	**(1.10–1.28)**	**(1.25–1.51)**	**(1.16–1.96)**	**(1.41–1.74)**	**(1.18–1.44)**
Russian Federation	0.95	**0.97**	**1.17**	**1.25**	**1.56**	**1.08**
	(0.88–1.03)	**(0.91–1.04)**	**(1.08–1.26)**	**(1.07–1.46)**	**(1.31–1.86)**	**(0.93–1.26)**
Ukraine	**1.12**	**1.12**	**1.09**	0.93	**1.67**	**0.99**
	**(1.02–1.24)**	**(1.03–1.21)**	**(1.00–1.18)**	(0.75–1.16)	**(1.52–1.84)**	**(0.91–1.08)**
**Northern European countries**						
Denmark	**1.46**	**1.27**	**1.58**	**1.28**	1.00	1.07
	**(1.31–1.62)**	**(1.17–1.38)**	**(1.43–1.75)**	**(1.13–1.46)**	(0.89–1.13)	(0.96–1.18)
Scotland	**1.15**	**1.08**	**1.47**	1.11	**0.86**	0.98
	**(1.05–1.27)**	**(1.00–1.16)**	**(1.35–1.59)**	(0.97–1.28)	**(0.77–0.95)**	(0.90–1.07)
Wales	**1.20**	**1.20**	**1.46**	0.97	**0.86**	0.99
	**(1.08–1.34)**	**(1.11–1.30)**	**(1.35–1.58)**	(0.78–1.17)	**(0.77–0.95)**	(0.90–1.09)
England	**1.25**	**1.14**	**1.44**	1.03	**0.85**	**1.13**
	**(1.13–1.39)**	**(1.05–1.24)**	**(1.33–1.56)**	(0.83–1.29)	**(0.75–0.95)**	**(1.01–1.28)**
***Estonia	1.10	1.03	**1.29**	1.23	-	1.05
	(0.98–1.23)	(0.93–1.15)	**(1.18–1.42)**	(0.89–1.70)		(0.95–1.15)
Finland	**1.17**	**1.10**	**1.65**	**1.30**	1.00	0.93
	**(1.09–1.26)**	**(1.02–1.20)**	**(1.53–1.77)**	**(1.04–1.61)**	(0.92–1.10)	(0.85–1.02)
****Greenland	1.19	1.05	**1.48**	**1.72**	-	-
	(0.85–1.69)	(0.76–1.45)	**(1.08–2.03)**	**(1.15–2.56)**		
Ireland	**1.28**	**1.20**	**1.55**	**1.46**	0.97	0.99
	**(1.15–1.43)**	**(1.10–1.32)**	**(1.39–1.72)**	**(1.18–1.81)**	(0.85–1.10)	(0.88–1.11)
Latvia	**0.84**	0.92	**1.21**	1.19	**1.64**	0.08
	**(0.75–0.94)**	(0.84–1.01)	**(1.11–1.32)**	(0.97–1.46)	**(1.45–1.84)**	(0.99–1.22)
**Northern European countries**						
Lithuania	**1.13**	**1.12**	**1.28**	**1.15**	**1.86**	**1.36**
	**(1.02–1.25)**	**(1.04–1.20)**	**(1.16–1.40)**	**(1.00–1.33)**	**(1.60–2.15)**	**(1.25–1.49)**
Sweden	**1.13**	1.08	**1.45**	0.88	1.06	1.09
	**(1.03–1.24)**	(0.99–1.19)	**(1.31–1.60)**	(0.74–1.04)	(0.94–1.20)	(0.98–1.21)
Norway	**1.44**	**1.31**	**1.78**	**1.39**	**1.11**	**1.20**
	**(1.28–1.61)**	**(1.21–1.42)**	**(1.63–1.93)**	**(1.18–1.64)**	**(1.01–1.23)**	**(1.10–1.36)**

***Wald test*, *p<0*.*05*:** *Adjusted by random effects of school and school class*.

*Missing class level 6289;

**Missing school level 4679

***Missing class level 3979;

****Missing class level 2257; Missing school level 859. *For each variables*, *the missing category is the reference; Wald test*, *p<0*.*0*.*5*. *United States*, *Slovenia*, *Czech Republic and Hungary are not considered because the association daily-daily in Breakfast Consumption is too low (<50%)*. *Belgium-Fl*, *Switzerland*, *Estonia and Greenland are not considered because missing information*.

**Trends from 2002 to 2010 in DBC.** Six countries showed an increase in DBC (Canada, Netherland, Macedonia, Scotland, Wales, England) from 2002. A significant decrease in DBC from 2002 was found in 11 countries (Belgium Fr, France, Germany, Croatia, Spain, Poland, Russian Federation, Ukraine, Latvia, Lithuania and Norway), while in 5 countries (Portugal, Denmark, Finland, Ireland, Sweden) no significant changes were seen (Table 3).

## Discussion

This study adds to the existing literature by being the first study to compare DBC trend data for 31 countries across two continents. It updates previous HBSC work [4] and adds new information by studying trends in DBC over nearly a decade in a multinational context using the same standardized methods. This study also adds to the literature on socio-economic correlates with DBC by studying associations based on pooled data covering three survey cycles of the HBSC study.

DBC ranged from 37.8% to 72.6% and DBC was most common among boys. DBC increased significantly in only six out of 19 countries from 2002 to 2010. The existing literature on changes over time in DBC are generally national and local level studies [20–22]. This is the first study to investigate trends across many countries. The differences in time trends in DBC across countries are not easily explained, but there may be some overarching/common factor(s) that require further investigation. We know that especially in Western countries, the availability of foods outside the home at all times of the day has increased [28]. This higher availability of foods especially snack foods outside the home might contribute to the decrease observed in DBC [29,30]. If left unchecked, these changes in food environments will continue to contribute to poor food habits, which over time, may further exacerbate rates of obesity and diabetes.

Public health agencies have tried to increase DBC. However, they have had less impact on low affluence families as our study and others demonstrate that DBC is generally higher among adolescents from high affluent families. Also in line with the existing literature, we found that in a majority of countries the proportion of adolescents consuming breakfast daily were generally higher in two-parent families [1, 6–13] and among boys [5]. The gender differences in breakfast consumption have been attributed to weight concerns among adolescent girls [2,3,31]. Additional factors that may impact on daily breakfast consumption include a limited knowledge about nutrition and health [32], lack of time to eat or prepare breakfast [33] and unavailability of foods for breakfast [2].

Generally the findings highlight the importance of the family environment for influencing the dietary behaviours of young people [18, 34–36]. Socialisation of health-related behaviours occurs within the family, with parents’ beliefs, attitudes and behaviours substantially affecting children's health behaviours [37]. Consistently, within the literature it is documented that parental eating behaviours are positively associated with both unhealthy [36] and healthy [14, 23, 38–45] dietary behaviours of children and adolescents. The differences in DBC observed among boys and girls however indicate that family-related processes affecting adolescents’ DBC may operate differently across gender.

When interpreting the results a number of study limitations should be considered. We investigated daily versus less than daily breakfast consumers and it could be argued that skipping breakfast on just one day does not have an effect on adolescent health, although others have found that breakfast consumption shows dose-response association with overweight [7]. Our measures of breakfast frequency has been validated and Kappa statistics comparing daily consumption of breakfast to diary measures in the Flemish population were fair for weekends (0.34) and moderate for weekdays (0.47). Further, the frequency measures have been validated in a Danish agreement study comparing daily consumption of breakfast with 24 hour recall during one week and the kappa statistics showed good agreement for the dichotomized item (0.62 weekdays and 0.46 weekend) [46].

The HBSC study is based on a frequency measure and defines breakfast as having more than a glass of milk or fruit juice. This measure precludes any assessment of the nutritional quality of the meal. Also, we did not distinguish between breakfast consumption during the school week and at weekends, which is pertinent when attempting to explain the rates of breakfast consumption over time and the settings where interventions are most needed. Indeed, there are numerous school-based breakfast programmes in operation across many countries although the results of our study should not be affected if meal programmes remained unchanged within countries. However, it was beyond the scope of this study to map school food programmes across countries. Yet our data, up to 2010 indicate that DBC remains low for many students. In part this could be due to less structured meal times at weekends for adolescents and their families.

Another issue relates to the use of FAS over time. There is a risk that the classification by FAS may not be uniform over time (e.g. by more families owing computers by increasing survey year) and it may be hypothesized that misclassifications of families in less affluent groups into more affluent groups may therefore increase over time. In such case there is a risk that the patterns of social inequality is increasingly being underestimated by each survey year.

This HBSC study offers an opportunity for cross-national and time trend analyses on adolescent health and the study of DBC covers three survey rounds spanning nine years across 31 countries. Other strengths include the large sample size and the representative samples of adolescents from 31 countries across two continents, completed by a standardized protocol for data collection ensuring internationally comparable data. DBC should be encouraged within the context of each country and family [17]. Increased attention to DBC is especially necessary during the transition from childhood to adolescence and especially in girls and young people from disadvantaged families. Both the home and school setting needs attention to reduce social inequalities in breakfast consumption. yield benefits on cognitive performance [10,11]. Further research should explore the countries that have experienced an increase in DBC over time and associated changes in policies, strategies and programmes. In particular, it would be valuable in a multilevel analytical design to describe and compare national characteristics and guidelines and their implementation taking cultural and normative practices into account.
